# Investigation of the Mechanical Properties of Composite Honeycomb Sandwich Panels after Fatigue in Hygrothermal Environments

**DOI:** 10.3390/polym16172497

**Published:** 2024-09-01

**Authors:** Ming Zhao, Haibo Jin, Zhaoxin Yun, Zhengwei Meng, Wei Zhang

**Affiliations:** 1 China Special Aircraft Research Institute, Jingmen 448035, China; zhaoming605@126.com (M.Z.);; 2 Department of Aircraft, Astronautics College of Aerospace Engineering, Nanjing University of Aeronautics, Nanjing 210001, China

**Keywords:** composite honeycomb sandwich panels, hygrothermal, compression performance, shear performance

## Abstract

Since carbon fibre composite sandwich structures have high specific strength and specific modulus, which can meet the requirements for the development of aircraft technology, more and more extensive attention has been paid to their residual mechanical properties after subjecting them to fatigue loading in hygrothermal environments. In this paper, the compression and shear characteristics of carbon fibre-reinforced epoxy composite honeycomb sandwich wall panels after fatigue in hygrothermal environments are investigated through experiments. The experimental results show that under compressive loading, the load required for the buckling of composite honeycomb sandwich wall panels after fatigue loading in hygrothermal environments decreases by 25.9% and the damage load decreases by 10.5% compared to those at room temperature. Under shear loading, the load required for buckling to occur is reduced by 26.2% and the breaking load by 12.2% compared to those at room temperature.

## 1. Introduction

Sandwich composites have the advantages of a light weight, high specific stiffness, good stability, heat insulation, and good corrosion resistance [1,2,3,4]. Therefore, they have been widely used in aerospace, marine, radar, and other military fields. However, the performance and service life of sandwich composites are affected by the environment (e.g., temperature, humidity, seawater, corrosive environments, etc.), which leads to a decrease in the mechanical properties of honeycomb sandwich composites [5,6,7,8,9]. In addition, sandwich constructions are finding increasing use in civil engineering applications, owing to their advantages over traditional materials [10,11]. Although the commonly used foams are lightweight closed-cell materials with a small moisture absorption rate, the performance of the product will also be degraded after moisture absorption [12,13,14,15], so the environmental resistance has become a basic requirement for the selection of composites for sandwich structures.

The mechanical properties of composite sandwich structures depend on the material properties of the composite panels and cores as well as their bonding properties. Scholars have thoroughly studied the mechanical properties and damage modes of honeycomb sandwich structures [16,17,18,19,20,21,22,23,24]. Paik et al. [20] investigated the effect of varying the thickness of the honeycomb core on the improvement of the compressive strength of sandwich panels by changing the thickness of the honeycomb core sub-walls. Gdoutos et al. [22] analysed and predicted the failure modes of honeycomb sandwich panels and foam sandwich panels in comparison with each other. In the case of lateral compression loading, the foam sandwich panel undergoes damage in the form of waves, while the honeycomb sandwich panel undergoes damage in the core layer. Kwon et al. [23] analysed the failure modes of honeycomb sandwich panels and foam sandwich panels, and the failure mode of honeycomb sandwich panels was predicted. Grediac [24] calculated the transverse shear modulus of honeycomb sandwich panels by performing finite element studies on representative unit cells. Three cell geometries were investigated and the effect of thickness on the shear modulus and the uniformity of the shear stress field was discussed.

Composite structures were often subjected to cyclic loading under various temperatures and humidity conditions, and the coupling of temperature and humidity can cause material degradation and the formation of hygrothermal stresses within the material. A series of studies have been carried out on the hygrothermal resistance of composites [25,26,27,28,29,30,31,32,33,34,35]. David et al. [32] investigated the changes in the inter-facial fracture strength of PVC foam sandwich composites after treatment under three different conditions, and the results showed that after the hygrothermal treatment, the fracture strength of the samples decreased to different degrees. Manjesh et al. [33] investigated the mechanical changes in Pu foam sandwich composites under hygrothermal conditions and found that the compression properties as well as the bending properties of the samples decreased to different degrees after hygrothermal treatment. Katzman et al. [34] sought to simulate the diffusion of water molecules in the sandwich composites, and the results showed that the diffusion constants of the water molecules in the foam core were much higher than the diffusion coefficients in the panels. Rao [35] investigated the hydrothermal aging resistance and flexural behaviour of glass fibre-reinforced polymer (GFRP) composites at different temperatures and exposure times. The results showed that moisture absorption and temperature effects lead to strength degradation during the life cycle of GFRP composites exposed to water and temperature for a long time.

In fatigue-damaged composite honeycomb wall panels in hygrothermal environments, a wide variety of internal damage occurs due to the combined effects of hygrothermal and fatigue loading. Their residual mechanical properties, as well as their damage modes, should be simultaneously characterized by accurate analytical methods. However, considering the effects of the hygrothermal environment and fatigue loading, existing research works have not investigated the residual properties of composite honeycomb wall panels. In this study, composite honeycomb sandwich wall panels were prepared using carbon fibre-reinforced epoxy resin composite panels. In order to investigate the moisture absorption rate as well as the mechanical properties of composite honeycomb sandwich wall panels under limiting conditions, the specimens were subjected to a hygrothermal environment in the course of the experiments to investigate the moisture absorption characteristics of the composite honeycomb sandwich wall panels, and the residual compression and shear properties of the specimens after fatigue loading in the hygrothermal environment were also measured. A concise overview of the materials utilized, the fabrication method, and the experimental setup is provided in Section 2. Section 3 presents the experimental test results. Finally, the conclusions are presented in Section 4.

## 2. Specimen and Experimental Setup

### 2.1. Specimen

**Figure 1:**
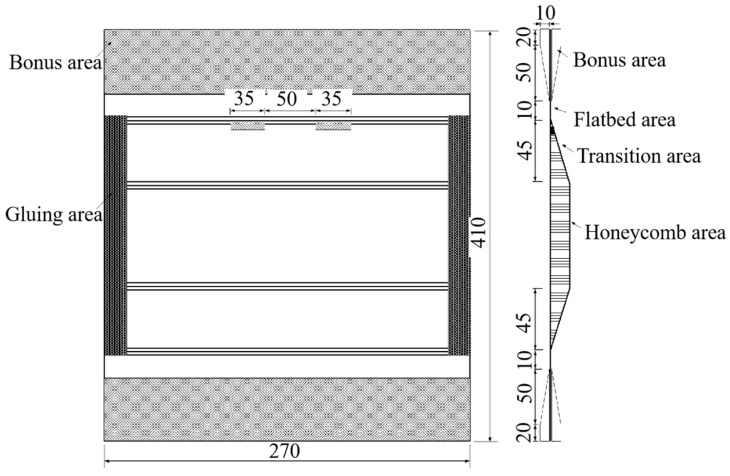
The structure of the compression specimen.

In the composite honeycomb wall plate compression fatigue specimen, as shown in Figure 1, the honeycomb core lattice direction was along the compression direction, and the specimen was 410 mm long and 270 mm wide. The sides of the top and bottom ends were clamped with bevelled aluminium blocks as the compression end faces of the test piece, which were glued to the composite honeycomb wall plate and then connected with a row of bolts. The left and right sides of the specimen were glued as the knife-edge clamping surface of the test piece. The specimen was made of T800 carbon fibre/epoxy composite laminate produced by Suzhou Lianqiao Tianyi Composite Technology Co. (Suzhou, China). The inner and outer panels and honeycomb core were co-cured and formed, the honeycomb tips of the wall panels were filled with foam adhesive J-118 (Shanghai Shibang Industry Co., Shanghai, China), and the panels and honeycomb core were glued with strip core adhesive J-116A-δ0.35 (Shanghai Xinmu Ming Co., Shanghai, China). The stacking sequences and dimensions of the inner and outer panels and honeycomb core are shown in Table 1.

**Figure 2:**
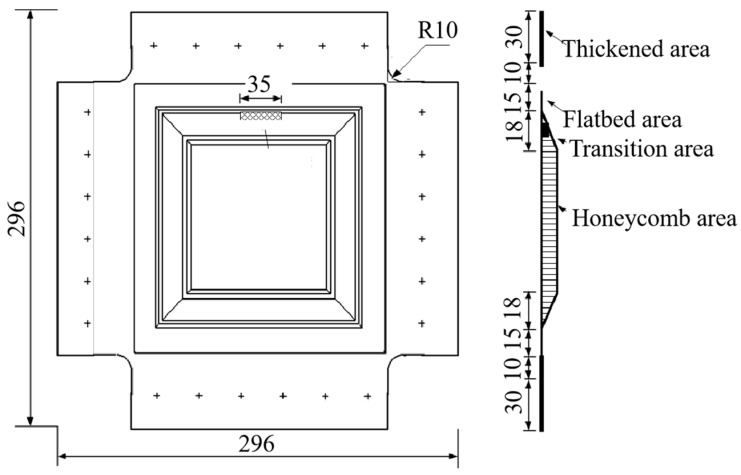
The structure of the shear specimen.

The composite honeycomb wall plate shear fatigue specimen, as shown in Figure 2, had a square structure and a length of 296 mm on each side. The four side laminates of the specimen were partially thickened to serve as the clamping ends, which were bolted together with the fixture. The inner and outer panels and honeycomb core were co-cured and formed, the honeycomb tips of the wall panels were filled with foam adhesive J-118, and the panels and honeycomb core were glued with strip panel core adhesive J-116A-δ0.35. The stacking sequences and dimensions of the inner and outer panels and honeycomb core are shown in Table 2.

### 2.2. Experimental Setup

#### 2.2.1. Hygrothermal Aging Test

The specimens were placed in the temperature of 70 °C and relative humidity of 85% of the hygrothermal test chamber for moisture absorption treatment and weighing test records (for a moisture absorption process with reference to the standard ASTM D522) [36]. With two successive changes in the weight of less than 0.05% of the initial weight, the specimen reaches the equilibrium of moisture absorption.

(1) $$\frac{W_{i}-W_{i-1}}{W_{b}}<0.0005$$

where $W_{i}$ is the weight at the current time;${\;W}_{i-1}$ is the weight at the previous time; and $W_{b\;}$ is the weight before hygroscopic wetting.

#### 2.2.2. Compression Test

**Figure 3:**
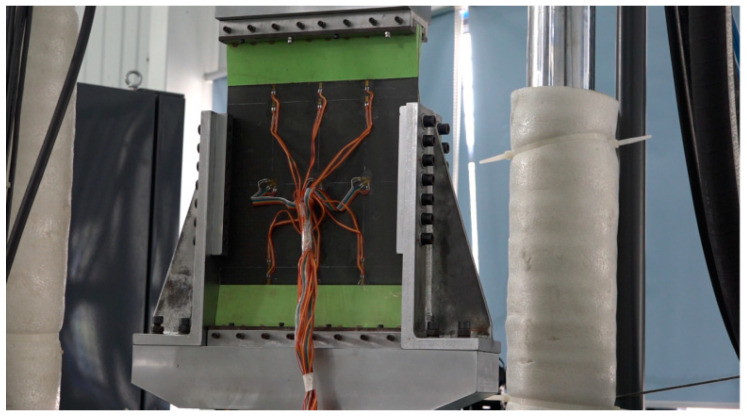
Compression test installation.

Quasi-compression experiments were used to investigate the residual compressive properties of the composite honeycomb wall panels after axial fatigue. The equipment used for the experiments was an MTS fatigue testing machine (Meters Industrial Systems, Eden Prairie, MN, USA) with a range of 250 KN. The fatigue test was conducted firstly by applying an equal-amplitude fatigue load. The cyclic load was 661 N~6610 N, the cycle number was 1 million times, the loading frequency was 5 Hz, and the loading waveform was sinusoidal. The residual compression test was carried out at the end of the fatigue test. As shown in Figure 3, a special fixture was used to mount the specimen on the fatigue testing machine, and the loaded end of the specimen and the honeycomb isostatic zone were clamped with a knife edge to simulate the support conditions of a simple support. The centre of the loaded end of the specimen was marked out first during mounting and ensured to be in line with the loaded centre.

#### 2.2.3. Shearing Test

**Figure 4:**
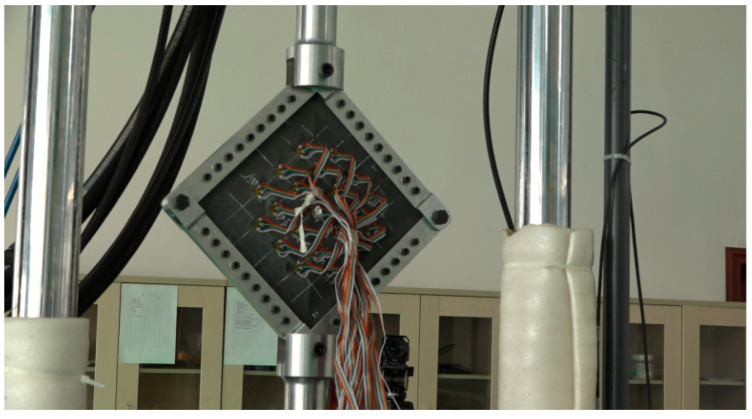
Shear test installation.

The residual shear properties of the composite honeycomb wall panels after fatigue were investigated using shear experiments. The equipment used for the experiments was an MTS fatigue testing machine with a range of 250 KN. The fatigue tests were conducted firstly by applying an equal-amplitude fatigue load. The cyclic load was 939 N~9386 N, the number of cycles was 1 million, the loading frequency was 5 Hz, and the loading waveform was sinusoidal. At the end of the fatigue test, a residual shear test was carried out. As shown in Figure 4, a special fixture was used to mount the specimen on the fatigue testing machine, and a row of connecting bolts was used on all sides of the specimen to fix it with the test fixture, and the shear load was applied by means of diagonal stretching.

### 2.3. Measurement Programme

**Figure 5:**
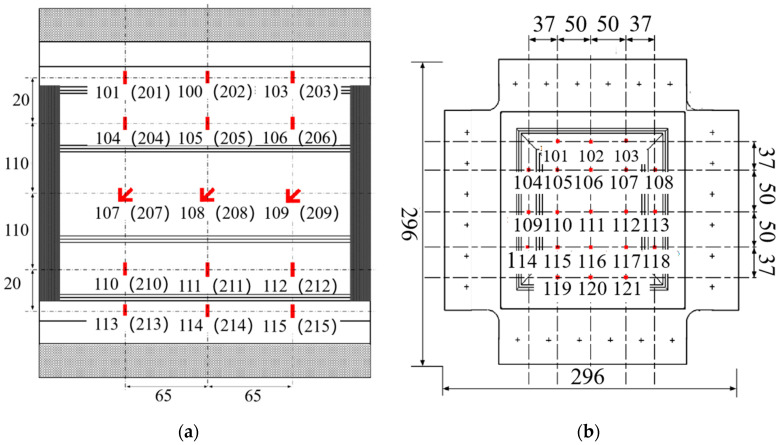
The strain gauge position: (**a**) compression test; and (**b**) shear test.

The compression specimen strain gauges’ paste position and number are shown in Figure 5a, showing the outer surface of the number from the beginning of 1, single-sided arrangement of three columns of five rows of strain gauges, symmetrical arrangement of the inner and outer surfaces, a total of 24 single-piece strain gauges and 6 pieces of flower strain gauges. Among them, 101–115 were pasted on the outer surface of the test piece and 201–205 were pasted on the inner surface of the test piece. Here, 107, 108, 109, 207, 208 and 209 were flower strain gauges. The shear specimen strain gauges’ paste location and quantity are shown in Figure 5b, showing the outer surface from the beginning of 1 sort, single-sided arrangement of five columns and five rows of strain gauges, symmetrical arrangement of the inner and outer surfaces, and a total of 42 pieces of flower sheet. Among them, 101–121 were pasted on the outer surface of the test piece and 201–221 were pasted on the inner surface of the test piece.

## 3. Experiment Results

### 3.1. Moisture Absorption Characterization

**Figure 6:**
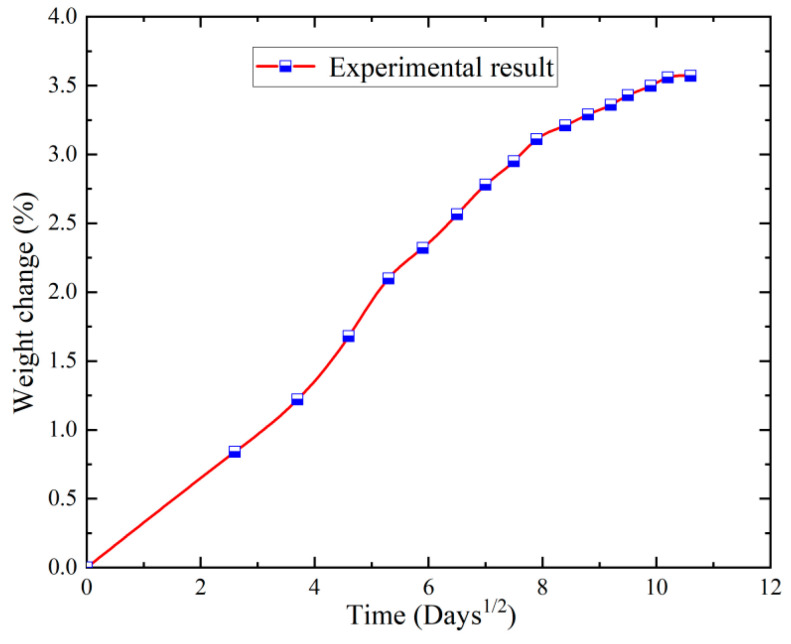
The moisture absorption process of the composite honeycomb sandwich wall panels.

The interaction of these two mechanisms was balanced due to the fact that the composites were not only hygroscopic during hygrothermal aging but also the weight of the composite was reduced by the release of water molecules. Figure 6 shows the process of water absorption of the carbon fibre composite honeycomb sandwich wall panels in a hygrothermal environment, which increases and saturates with the aging time and reaches 3.5% at 2400 h. Thereafter, with the extension of the aging time, the water absorption no longer increases. This is because the moisture absorption behaviour of epoxy tree-based composites during hygrothermal ageing mainly consists of the diffusion of water molecules in the resin matrix and the aggregation of water molecules in the pores, cracks and defects at the interface of the fibres and resin. 

### 3.2. Compression Test Results

**Figure 7:**
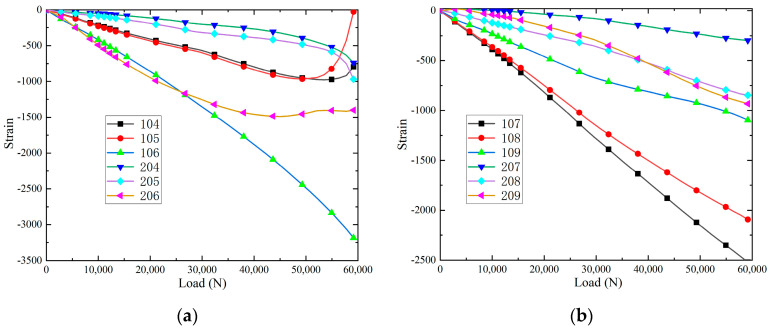
The strain-load curves: (**a**) upper part; and (**b**) lower part.

The strain-load relationship of the specimens at different positions at room temperature is shown in Figure 7. It can be found that when loaded to 27,000 N, the slopes of the load-strain curves at the strain measurement points of 104, 105 and 106 (upper part) gradually decrease, indicating that there are signs of buckling in this region, and that these buckling regions occur with the presence of debonding damage between the panel and the core layer, which leads to local buckling in the damaged region. When loaded to 50,000 N, the strain-load curves at 104, 105 and 106 change from a linear increase to a non-linear deviation, and the strain-load curves at 104 and 105 are turned, indicating that overall buckling deformation of the panels and the core layer occurred, and that a large number of cracks were generated in both the panels and the core layer. In addition, intermittent peeling and crunching sounds are emitted, which are due to the extension of the flexural wave to the edge of each panel and core layer, resulting in further extension of the debonding damage between each panel and core layer, and a shift in the strain-load curves at 104 and 105, which indicates that the panels and the core layer underwent an overall flexural deformation and that a large number of cracks were generated in the panels and the core layer. The strain load curves at the 110, 111 and 112 (lower part) strain note locations changed from linear growth to non-linear deviation at a load of 35,000 N, which proved that only buckling of the panels and the core layer occurred at the lower end of the test specimen, and no damage was produced. When loaded to 570,000 N, the debonding damage between the panel/core layer was fully extended, and the upper end of the test specimen produced a large number of cracks and a large amount of buckling, and the loss of load carrying capacity is shown in Figure 9a.

**Figure 8:**
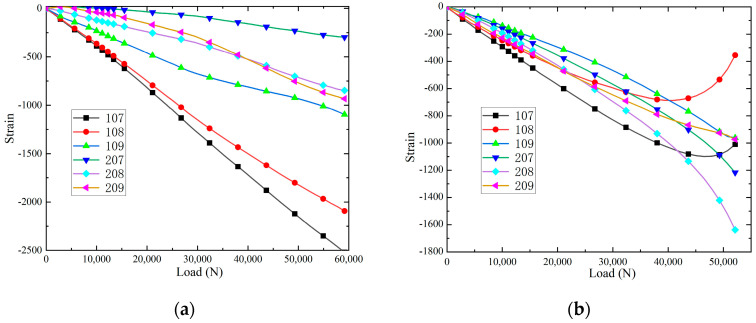
The strain-load curves: (**a**) upper part; and (**b**) lower part.

**Figure 9:**
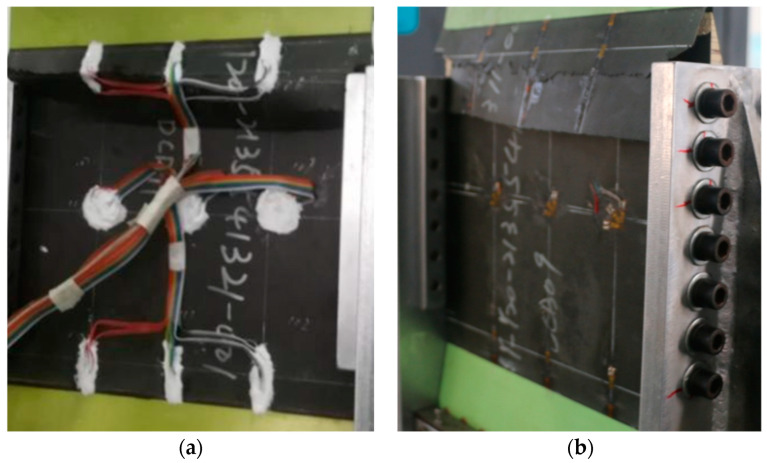
The forms of damage to the specimens: (**a**) room temperature; and (**b**) hygrothermal temperature.

The strain-load curves of the specimens at different locations in the hygrothermal environment are shown in Figure 8. When loaded to 20,000 N, the slopes of the load–strain curves at the strain measurement points of 104, 105 and 106 gradually decrease, resulting in local buckling in the panel region. When loaded to 40,000 N, the strain-load curves at 104, 105, 106, 205 and 206 turn, indicating that the panel and core layer underwent overall buckling deformation and a large number of cracks were generated in the panel and core layer. In addition, the strain-load curves at 110, 111, 112, 210, 211, and 212 strain notation positions change from linear growth to non-linear deviation at a load of 25,000 N, which proves that only the buckling phenomenon of the panel and the core layer occurs at the lower end of the test piece, and a small amount of crack damage is produced. When loaded to 420,000 N, the strain-load curves of 107 and 108 shift, indicating that a large number of cracks were produced in the panels and core layer. When loaded to 510,000 N, the debonding damage between each panel/core layer is fully extended, and the upper end of the test piece produces a large number of cracks and develops a large amount of buckling, and it loses its load carrying capacity, as shown in Figure 9b. In addition, it can be seen that the load ratio required for buckling to start to occur in hygrothermal aging specimens decreases by 25.9%, the load ratio required for specimens to break load decreases by 10.5%, and the load ratio required for strain-load curves to produce non-linear deviations decreases by 20% compared to room temperature.

### 3.3. Shearing Test Results

**Figure 10:**
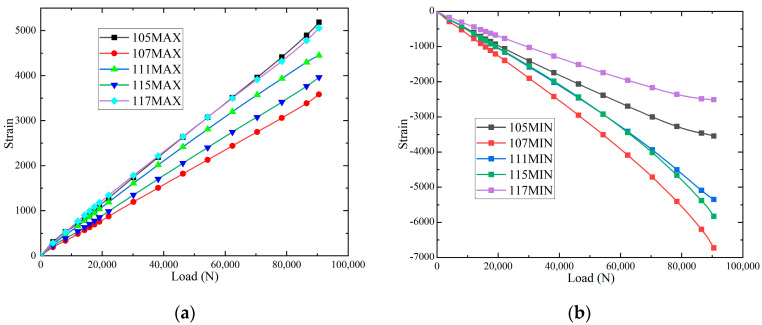
The strain-load curves: (**a**) maximum principal strain; and (**b**) minimum principal strain.

**Figure 11:**
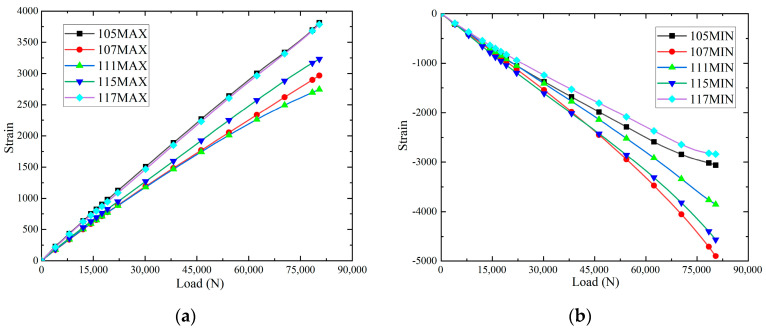
The strain-load curves: (**a**) maximum principal strain; and (**b**) minimum principal strain.

**Figure 12:**
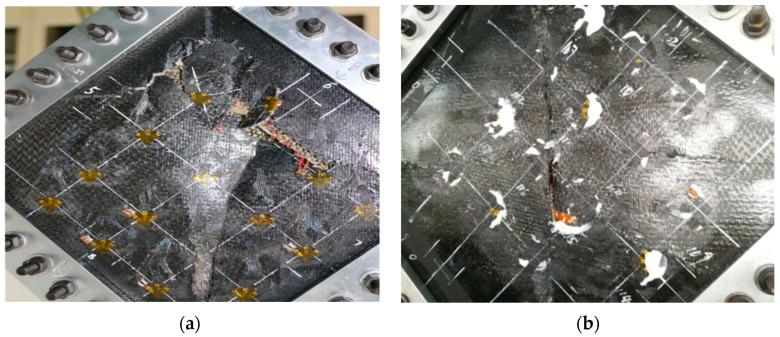
The forms of damage to the specimens: (**a**) room temperature; and (**b**) hygrothermal temperature.

The maximum principal strain-load curves and minimum principal strain-load curves, and of the specimens at different positions at room temperature, are shown in Figure 10. It can be seen that the slopes of the load–strain curves are significantly larger than those of 115 and 117 when loaded to the strain measurement points 105, 111 and 117, and the slopes of the load–strain curves are larger than those of 111 at the strain measurement points 105 and 117, which indicates that the damage will be produced at the earliest time at the strain measurement points 105 and 117. Because the positions at 105, 111 and 117 are on the loading centre line, these areas will be the first to produce damage and destroy. When loaded to 61,000 N, the slopes of the strain-load curves of 105, 115 and 117 change slightly, indicating that the panels and the core layer underwent overall flexural deformation. Loading to 90,000 N, the debonding damage between each panel/core layer is fully extended, and 105, 111 and 117 are at the loading centre line to produce cracks, and the lost test piece de-loading capacity is shown in Figure 12a. The maximum principal strain-load curves and minimum principal strain-load curves of the specimens at different locations in the hygrothermal environment are shown in Figure 11. When loaded to 45,000 N, the slopes of the strain-load curves of 105, 107, 115 and 117 change slightly, indicating that the panels and the core layer underwent overall buckling deformation. Loading to 79,000 N, the debonding damage between each panel/core layer is fully extended, and 105, 111 and 117 are at the loading centre line to produce cracks and lose the test piece de-loading capacity, as shown in Figure 12b. In addition, it is observed that the load required for the hygroscopic saturated specimen to start buckling decreases by 26.2% compared to room temperature, and the load required for the destructive load of the test specimen decreases by 12.2%.

## 4. Conclusions

In this paper, an experimental study was carried out to investigate the mechanical properties and the effect of the failure modes of epoxy resin carbon fibre composite honeycomb sandwich wall panels after fatigue under humid-heat aging. Firstly, the moisture absorption characteristics of the carbon fibre composite honeycomb sandwich wall panels were analysed. Then, the composite honeycomb panel was subjected to one million fatigue tests and its compression and shear properties after fatigue were investigated and the effect of humid-heat aging on its remaining mechanical properties after fatigue was analysed. The specific conclusions are as follows:

(1) For the epoxy resin carbon fibre composite honeycomb sandwich wall panel, with the extension of the wet-heat aging time, its water absorption rate increases and tends to be saturated. In the aging of 2400 h, its water absorption rate reaches 3.5%. Thereafter, with the extension of the aging time, the water absorption rate no longer increases.

(2) Under compressive loading, the load required for the onset of buckling decreases by 25.9%, the load required for specimen rupture decreases by 10.5%, and the load required to produce a non-linear deviation in the strain-load curve decreases by 20% after the fatiguing of hygroscopically saturated epoxy carbon fibre composite cellular sandwich wall panels, as compared to those at room temperature.

(3) Under shear loading, the load required for the onset of buckling after fatigue of the moisture-absorbent saturated epoxy resin carbon fibre composite honeycomb sandwich wall panels is reduced by 26.2% and the load required for the rupture of the test piece is reduced by 12.2% in comparison to the ambient temperature.

## Figures and Tables

**Table 1:** The stacking sequences and thickness direction dimensions of the compression specimen.

Position	Thickness/mm	Stacking Sequences
Flatbed area	2.67	[(±45)/0/(±45)/0/0/+45/90/−45/0]s
Transition area	0.835 + 15.0 + 1.085	[(±45)/0/(±45)/0/0/NRH-2.0-48-δ15/90/+45/0/0/(±45)/0/(±45)]
Honeycomb area	0.585 + 15.0 + 0.46	[(±45)/0/(±45)/NRH-2.0-48-δ15/(±45)/(±45)]

**Table 2:** The stacking sequences and thickness direction dimensions of the shear specimen.

Position	Thickness/mm	Stacking Sequences
Thickened area	3.59	[(±45)/0/(±45)/90/(±45)/0/(±45)/0/45/90/−45]s
Flatbed area	2.17	[(±45)/0/(±45)/0/45/90/−45]s
Transition area	0.835 + 6.0 + 1.335	[(±45)/0/(±45)/0/45/NRH-2.0-48-δ6/90/−45/−45/90/45/0/(±45)/0/(±45)]
Honeycomb area	0.585 + 6.0 + 0.585	[(±45)/0/(±45)/NRH-2.0-48-δ6/(±45)/0/(±45)]

## Data Availability

The original contributions presented in the study are included in the article, further inquiries can be directed to the corresponding authors.
